# Unusual case of recurrent thigh lump in a girl: a case report

**DOI:** 10.1186/1752-1947-8-245

**Published:** 2014-07-06

**Authors:** Lasitha Samarakoon, Tharanga Fernanado, Eshwari Liyanage, Himaru Wirithamulla, Kaluarachige Sunil Perera

**Affiliations:** 1 General Surgical Unit, National Hospital of Sri Lanka, Colombo, Sri Lanka

**Keywords:** Lipofibromatosis recurrence

## Abstract

**Introduction:**

Lipofibromatosis is a rare fibro-fatty tumour with a predilection to involve distal extremities. It has only recently been described as a distinctive clinicopathologic entity, and subsequently only a few cases have been published in the literature. To address the clinicopathologic significance of this rare entity, we here describe a case of lipofibromatosis occurring on the left thigh of a Sri Lankan girl who developed a recurrence following excision.

**Case presentation:**

A 15-year-old previously healthy girl of Sri Lankan ethnicity presented with a painless progressively enlarging mass in her left thigh. Magnetic resonance imaging of her thigh lump, revealed a septated mass arising from subcutaneous tissue of anterolateral and medial aspects of her thigh. Histological assessment revealed evidence of lipofibromatosis, and the lesion was excised followed by split-skin grafting. She presented again with a local recurrence at the same site.

**Conclusions:**

Adequate surgical excision leads to complete cure of this benign lesion, but recurrences are common following incomplete excision. Therefore awareness among clinicians of this rare entity is vital in offering the best possible care to the patients.

## Introduction

Lipofibromatosis is a recently described soft tissue tumour of obscure aetiology [1]. We present a case of a Sri Lankan girl presenting with a slow growing, painless mass in her left thigh, with histological evidence of lipofibromatosis.

## Case presentation

**Figure 1:**
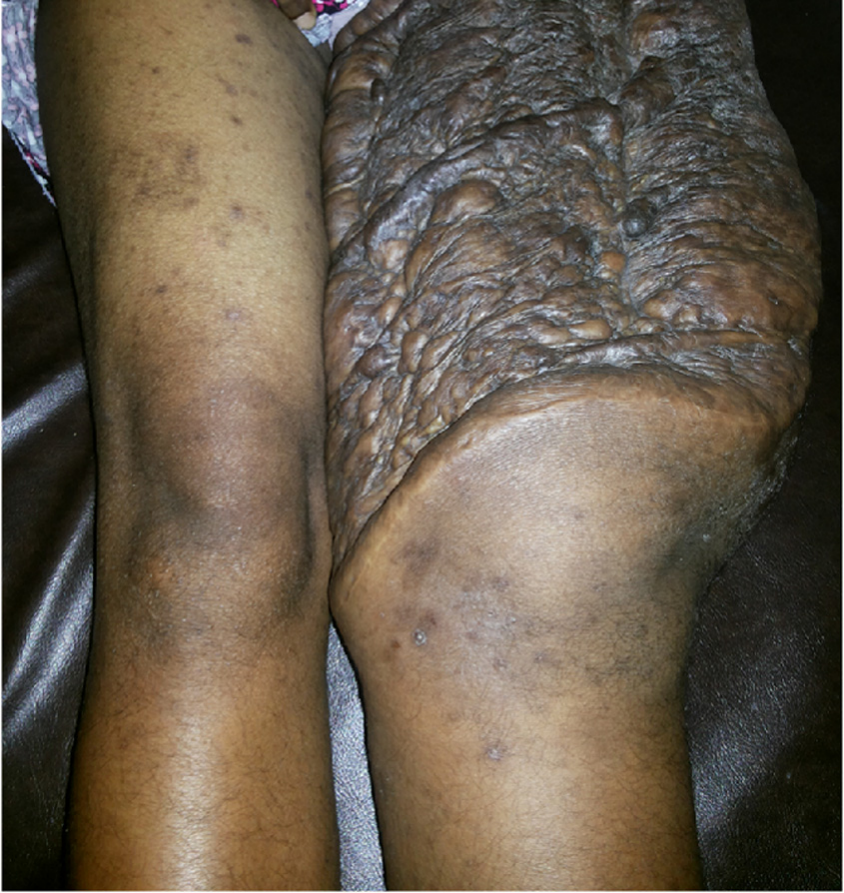
The patient presented with a painless progressively enlarging recurrent lump following excision on her left thigh, free of any deep attachments or neurovascular compromise.

**Figure 2:**
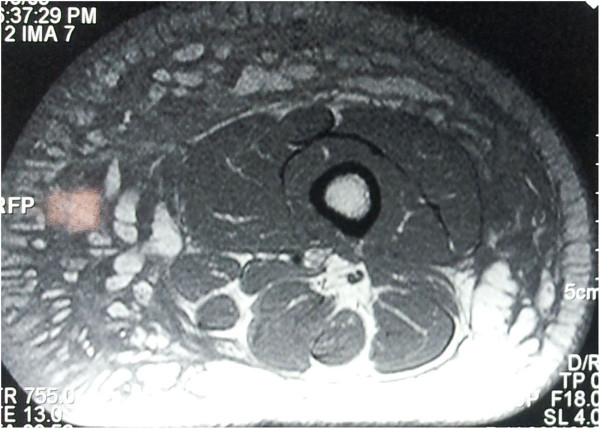
Magnetic resonance imaging scan of the thigh showing thick septate mass arising from subcutaneous tissue of anterolateral and medial aspects of the thigh without any contrast enhancement-transverse section.

**Figure 3:**
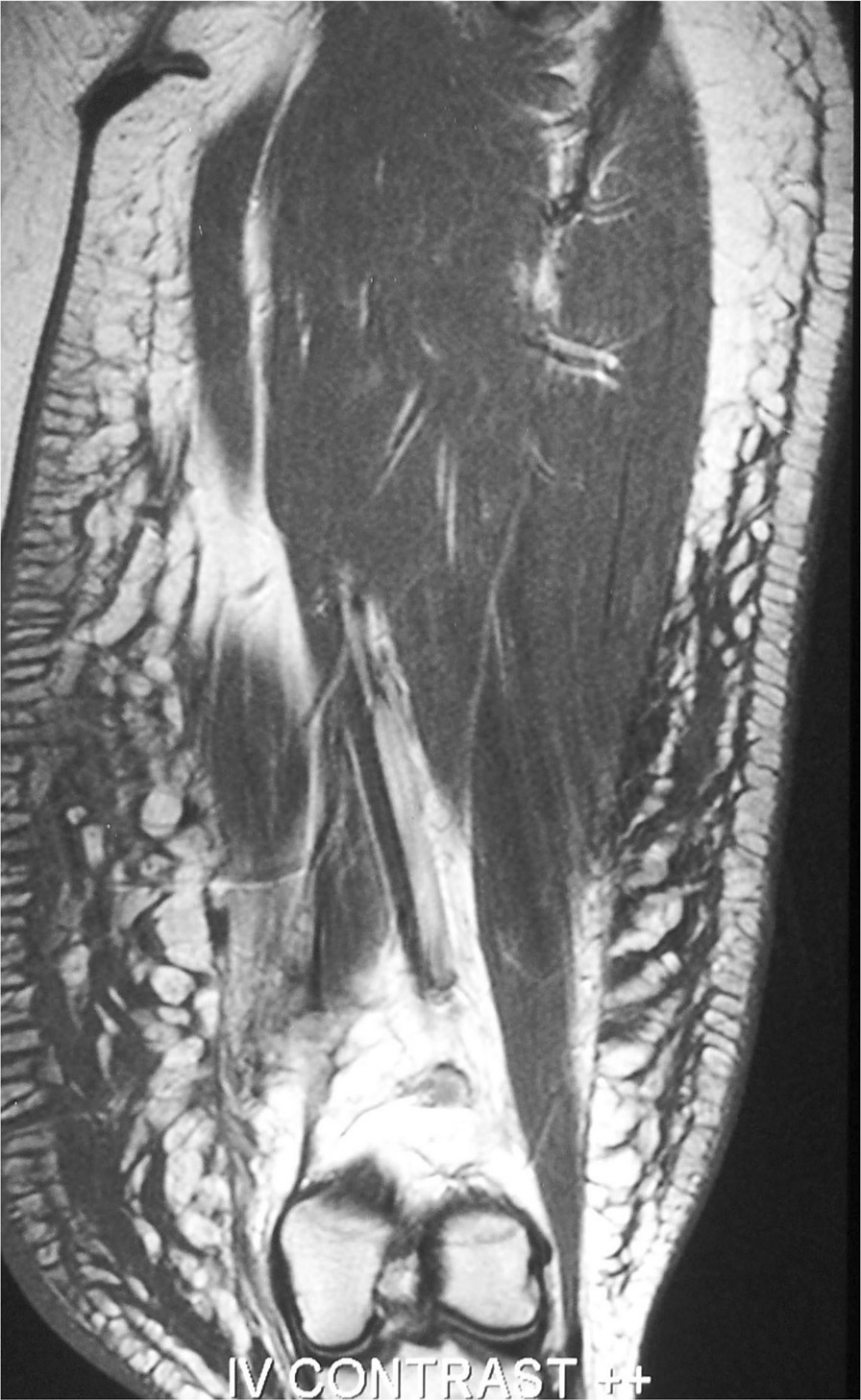
Magnetic resonance imaging of the thigh mass showing a sagittal section.

A 15-year-old girl of Sri Lankan ethnicity presented with a painless progressively enlarging thigh lump for a period of 2 years. She was otherwise healthy. On examination a non-tender mass of soft consistency was noted arising from the anterior compartment of her thigh. There were no neurological and vascular compromises, and the lump was free of any deep attachments. Both hip and knee joints were clinically normal. Initial haematological investigations were within normal range.Following initial investigation, she underwent a magnetic resonance imaging (MRI) scan of her left thigh. A 6cm thick septate mass arising from subcutaneous tissue of anterolateral and medial aspects of her thigh was noted. Contrast enhancement following intravenous gadolinium was not noted. Underlying muscle and bone were normal (Figures 1 and 2).She underwent incisional biopsy under local anaesthesia subsequently. Histological diagnosis of lipofibromatosis of her left thigh was reached. Features of malignancy were not noted. The lesion was later excised and split-skin grafting was done. She was followed up as an out-patient and she developed a local recurrence after 10 months at the same site (Figure 3).

## Discussion

Lipofibromatosis is a rare benign neoplasm usually occurring in childhood. Although it was previously designated as infantile fibromatosis of non-desmoid type [2], Fetsch *et al*. proposed that it should be classified as a separate entity as lipofibromatosis because it has distinct clinical and histological features [1].

Lipofibromatosis commonly presents as a painless slow growing mass affecting distal extremities, such as hands or feet, and, as was the case in our patient, in proximal limbs, trunk or even head and neck regions [3-5]. Aetiology of the tumour remains elusive to date [1].

On histological examination the tumour is mainly composed of mature adipose tissue [5]. Abundant fat tissue is separated by septa containing spindle fibroblast-like cells. They may have a primitive fibroblastic appearance; thus the lesion may mimic fibrous hamartoma of infancy.

Usually the mitotic rate is low and there is minimal cellular atypia [1]. Unlike fibromatosis, lipofibromatosis generally does not demonstrate solid, sheet-like fibrous growth.

Diagnosis can be made with distinctive histopathologic features, although immunohistochemical studies are an invaluable aid in doubtful cases [1,2]. Commonly, lipofibromatosis exhibits staining of the spindle cells with CD99, smooth muscle actin, BCL-2 and less commonly positivity for S-100 protein and epithelial membrane antigen. They stain typically negative to desmin [1,2,6]. Nuclear staining for beta-catenin by immunohistochemistry is commonly used to aid the diagnosis of fibromatoses, more so when the differential diagnosis includes other spindle cell tumours. In one study by Carlson and Fletcher, it was concluded that nuclear staining for beta-catenin is supportive, but not definitive, of the diagnosis of desmoid fibromatosis as beta-catenin negativity does not preclude the diagnosis of fibromatosis [7].

Imaging is an invaluable adjunct to diagnosing these rare tumours. On MRI, the detection of fat within the tumour is a valuable distinguishing feature between lipofibromatosis and the other soft tissue fibrous tumours [5]. Radiological differential diagnosis includes lipofibromatosis hamartoma and Proteus syndrome.

Lipofibromatous hamartoma (macrodystrophia lipomatosa) of the nerves is a slow growing mass of fibro-fatty tissue surrounding and infiltrating major nerves. It is associated with macrodactyly and bone overgrowth [8].

The clinical criteria for Proteus syndrome include the presence of a connective tissue nevus, ovarian cystadenomas or parotid adenomas as well as vascular malformations [9].

Although it commonly presents as a painless slow growing mass, compression or entrapment of vessels, nerves and muscle has been reported previously with lipofibromatosis [2].

Lipofibromatosis is notorious for local recurrence, as was the case with our patient. A high recurrence rate was noted following incomplete resection [1]. Congenital onset, male gender and high mitotic count appear to be other risk factors for recurrence.

## Conclusions

In conclusion, we propose that this rare and elusive clinical entity should be considered by clinicians and pathologists when encountering a soft tissue tumour in children and young adults. Although the number of documented cases is increasing, we believe that there is still insufficient clinical experience and lack of awareness about its existence. Since adequate surgical excision leads to complete cure of this benign lesion, awareness among clinicians is vital in offering the best possible care to the patients.

## Consent

Written informed consent was obtained from the patient’s legal guardian(s) for publication of this case report and any accompanying images. A copy of the written consent is available for review by the Editor-in-Chief of this journal.

## Abbreviations

MRI: Magnetic resonance imaging.

## Competing interests

The authors declare that they have no competing interests.

## Authors’ contributions

LS (registrar in surgery), TF and EL (intern medical officers) gathered data. LS prepared the manuscript. HW (senior registrar in surgery) and KSP (senior consultant in general surgery) made critical revisions to the original manuscript. LS, HW and KSP were responsible for overall surgical care of the patient. All authors read and approved the final manuscript.
